# Protective Effects of Hydrogen Sulfide in the Ageing Kidney

**DOI:** 10.1155/2016/7570489

**Published:** 2016-11-01

**Authors:** Cui-Lan Hou, Ming-Jie Wang, Chen Sun, Yong Huang, Sheng Jin, Xue-Pan Mu, Ying Chen, Yi-Chun Zhu

**Affiliations:** Research Center on Aging and Medicine, Fudan University, Shanghai Key Laboratory of Bioactive Small Molecules, Department of Physiology and Pathophysiology, Shanghai Medical College, Fudan University, Shanghai, China

## Abstract

*Aims*. The study aimed to examine whether hydrogen sulfide (H_2_S) generation changed in the kidney of the ageing mouse and its relationship with impaired kidney function.* Results*. H_2_S levels in the plasma, urine, and kidney decreased significantly in ageing mice. The expression of two known H_2_S-producing enzymes in kidney, cystathionine *γ*-lyase (CSE) and cystathionine-*β*-synthase (CBS), decreased significantly during ageing. Chronic H_2_S donor (NaHS, 50 *μ*mol/kg/day, 10 weeks) treatment could alleviate oxidative stress levels and renal tubular interstitial collagen deposition. These protective effects may relate to transcription factor Nrf2 activation and antioxidant proteins such as HO-1, SIRT1, SOD1, and SOD2 expression upregulation in the ageing kidney after NaHS treatment. Furthermore, the expression of H_2_S-producing enzymes changed with exogenous H_2_S administration and contributed to elevated H_2_S levels in the ageing kidney.* Conclusions*. Endogenous hydrogen sulfide production in the ageing kidney is insufficient. Exogenous H_2_S can partially rescue ageing-related kidney dysfunction by reducing oxidative stress, decreasing collagen deposition, and enhancing Nrf2 nuclear translocation. Recovery of endogenous hydrogen sulfide production may also contribute to the beneficial effects of NaHS treatment.

## 1. Introduction

Population ageing is a global phenomenon and exerts heavy demands on the healthcare system and society. The aged population, 65 years or older, will reach 1 billion people, accounting for 13% of the total worldwide population in 2030 [1]. Ageing is a natural process accompanied by gradual declining in physiological functions. Impaired renal function in ageing people is of great clinical relevance and usually associates with cardiovascular diseases and even mortality. The characteristics of the ageing kidney include nephrosclerosis, nephron hypertrophy, cortical volume reduction, and cyst formation [2]. On the other hand, the high frequency of underlying diseases among ageing people, such as concurrent diabetes, complicates the treatment of nephropathy. Therefore, understanding the process of kidney ageing might help to improve the quality of life of the ageing population and to provide precise treatment for senile nephropathy.

Hydrogen sulfide (H_2_S) is a gasotransmitter generated endogenously by cystathionine-*γ*-lyase (CSE), cystathionine-*β*-synthase (CBS), and 3-mercaptopyruvate sulfurtransferase (3-MST). H_2_S has diverse physiological functions such as relaxing blood vessels, lowering blood pressure [3, 4], antiapoptosis [5], anti-inflammation [6], and antioxidative stress [7]. In recent years, emerging studies have focused on the possibility of life span elongation by H_2_S. Miller and Roth firstly reported the regulatory role of H_2_S (50 ppm) in* C. elegans* ageing [8], and Wei and Kenyon recently confirmed this effect [9].

The beneficial effects of H_2_S on lifespan elongation involve both direct and indirect mechanisms. Some key regulatory molecules, such as Sirtuins [8] and Klotho [10], contribute to the direct effects of H_2_S, whereas the antioxidative nature of H_2_S protects the ageing heart or brain indirectly [11, 12]. Organ-specific mechanisms are of great clinical value for treating ageing-related diseases as well as pursuing healthy ageing. Our previous study showed that heart H_2_S levels in long-term fructose-fed ageing mice decreased from 0.020 *μ*mol/g protein to 0.013 *μ*mol/g protein, which may play some roles in the pathogenesis of diabetic cardiomyopathy [13]. The aim of this study was to investigate the endogenous production of H_2_S in the ageing kidney and the effect of chronic H_2_S supplements in protecting the kidney from ageing-related damage.

## 2. Materials and Methods

### 2.1. Animals and NaHS Administration

Eight-week-old male C57BL/6 mice were purchased from Department of Laboratory Animal Science of Fudan University and raised under controlled conditions (22 ± 2°C, 45–55% relative humidity, and 12 h dark-light cycle), with unrestricted access to diet and water until 16 months of age (old group). Another group of 8-week-old male C57BL/6 mice were purchased and raised until 3 months of age (young control group). We further divided the old mice into four groups: old control with normal saline, old with low-dose NaHS (10 *μ*mol/kg/day), old with medium-dose NaHS (50 *μ*mol/kg/day), and old with high-dose NaHS (100 *μ*mol/kg/day). The treatment, which consisted of intraperitoneal injection of NaHS or normal saline once a day, lasted for 10 weeks. All animal studies were approved by the Ethics Committee of Experimental Research, Fudan University Shanghai Medical College.

### 2.2. Metabolism and Biochemical Analyses

Mice were placed in metabolic cages (Tecniplast, Italy) separately for metabolism evaluation. After 3 days' acclimation, 24-hour water and food intake were measured and urine was collected. Glucose strips (OneTouch, Johnson) were used to determine fasting plasma glucose with blood collected from the tail vein before euthanasia. Plasma was obtained by centrifuging a blood sample at 3000 rpm, 4°C for 15 minutes (min). Plasma levels of creatinine (Crea), blood urea nitrogen (BUN), total cholesterol (CHOL), triglycerides (TG), low-density lipoprotein cholesterol (LDL-C), and high-density lipoprotein cholesterol (HDL-C) were determined by automatic biochemical analyser (Cobas 6000, Roche, Basel, Switzerland).

### 2.3. Detection of Reactive Oxygen Species (ROS) Levels

ROS levels in the kidney were measured using dihydroethidium (DHE) staining (Sigma-Aldrich) [14]. Briefly, DHE powder was dissolved in dimethyl sulfoxide and further diluted with phosphate-buffered saline (PBS) at 55°C until fully dissolution. Mice were injected with DHE solution (100 *μ*L, 27 mg/kg) maintained at 37–40°C and reinjected after 30 min. Eighteen hours later, mice were anesthetized and the kidney tissues were embedded in optimum cutting temperature compound (OCT). Mice kidney tissue sections (7 *μ*m) were obtained by using a frozen tissue slicer and observed under a laser confocal microscope (Zeiss LSM710) at the wavelength of 488/610 nm. Florescence values were normalized to the old groups.

### 2.4. Morphological and Histological Analyses

The kidney tissues were excised, fixed in 10% formalin, and embedded in paraffin. Kidney sections (4 *μ*m) were stained with Masson's, hematoxylin and eosin (HE), and TUNEL stain, according to the manufacturer's instructions. Renal pathological changes were observed under an optical microscope. Apoptosis was determined through TUNEL staining.

### 2.5. Measurement of H_2_S Levels and the Activity of H_2_S-Producing Enzymes

H_2_S levels in plasma, urine, and kidney tissues were determined as previously described [15]. The activity of CSE/CBS in the kidney tissues was measured by the method described by Tao et al. [16]. Briefly, 260 *μ*L homogenized kidney tissues were incubated together with 20 *μ*L L-cysteine (10 mmol/L) and 20 *μ*L pyridoxal-5′ phosphate (2 mmol/L) in an EP tube for 30 min at 37°C. Then 30 *μ*L supernatant obtained after centrifugation (10 min at 12000 rpm) was incubated with 80 *μ*L monobromobimane (MBB) for 40 min on a shaker at room temperature. Reaction was terminated by adding 20% formic acid and tested by gas chromatography-mass spectrometry (GC-MS). It should be borne in mind that this method can only test the overall activity of CSE and CBS because they share the same substrates.

### 2.6. Enzymatic Activity Assay

SIRT1 activity was determined by using the SIRT1 Fluorometric Drug Discovery Kit (BML-AK555-0001, Enzo) according to the manufacturer's protocol [17]. Briefly, for cell-free measurement of the reaction between H_2_S and recombinant SIRT1, 10 *μ*L SIRT1 protein (0.25 U) and 5 *μ*L NaHS (0, 12.5, 25, 50, and 100 *μ*mol/L) were incubated with 5 *μ*L substrate (0.25 mmol/L) and 5 *μ*L NAD (0.25 mmol/L) plus 25 *μ*L assay buffer. For tissue SIRT1 activity measurement, the reaction system contains 10 *μ*L kidney tissue homogenate, 5 *μ*L substrate (0.25 mmol/L), and 5 *μ*L NAD (0.25 mmol/L) plus 30 *μ*L assay buffer. Both reactions were carried out at 37°C for 40 min and stopped by addition of 1x Fluor de Lys® Developer II plus nicotinamide (50 *μ*L per well) (every 1 mL stop solution contains 760 *μ*L assay buffer, 40 *μ*L 50 mmol/L nicotinamide, and 200 *μ*L 5x Developer II). Fifteen mins later, fluorescence values were measured on a fluorometric reader (Synergy™ Mx, USA) with excitation at 360 nm and emission at 460 nm.

### 2.7. Western Blot Analysis

The kidney cytoplasm nuclear proteins were collected under the kit protocol (Nuclear and Cytoplasmic Protein Extraction Kit, Beyotime Biotechnology, Nanjing) and quantified using a BCA reagent (Shen Neng Bo Cai Corp, Shanghai). The proteins were resolved on a sodium dodecyl sulfate 10% polyacrylamide gel and transferred onto polyvinylidene fluoride membrane (Millipore, Bedford, MA, USA) and incubated with primary antibodies (1 : 1000 dilution) against Bcl-2, Bax, CSE, CBS, 3-MST, SIRT1 (Santa Cruz, CA, USA), Collagen I (Col I), Collagen III (Col III), Fibronectin (FN), SOD1, SOD2 (Abcam Company, USA), or Nrf2, HO-1 (Proteintech, China) at 4°C overnight. The blots were washed with phosphate buffer saline (TBST) for three times and then incubated with horseradish peroxidase-conjugated secondary antibodies for another 1 hour at room temperature. After washing, the blots were visualized by using chemiluminescent substrate (ECL). The densities of immunoblot bands were analysed using a scanning densitometer (model GS-800, Bio-Rad Laboratories, Hercules, CA, USA) coupled with Bio-Rad personal computer analysis software.

### 2.8. Statistical Analysis

Results are expressed as mean ± SEM. Statistical analysis was performed using SPSS software, version 21.0 (SPSS, Inc., Chicago, IL, USA). Comparisons among groups were performed by one-way ANOVA. Paired data were evaluated by two-tailed Student's *t*-test. Statistical significance was considered when *P* < 0.05.

## 3. Results

### 3.1. Hydrogen Sulfide Donor NaHS Has a Protective Effect in Ageing Mice Metabolism

**Figure 1:**
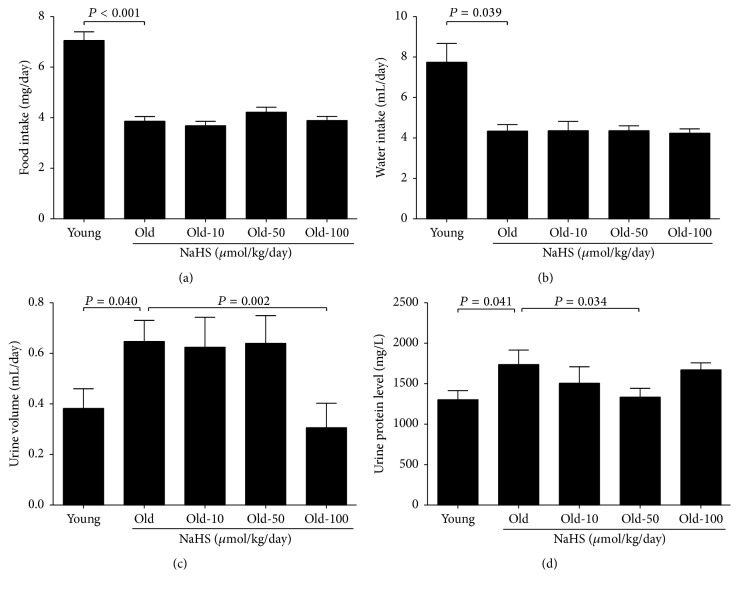
Twenty-four-hour metabolic characteristics of young and old mice. The old groups were treated with different doses of NaHS or saline as the control for 10 weeks. (a) Food intake. (b) Water intake. (c) Urine volume. (d) The level of urinary protein. Values are mean ± SE. *P* < 0.05 was considered significant (young groups, *N* = 12; old groups, *N* = 14).

**Figure 2:**
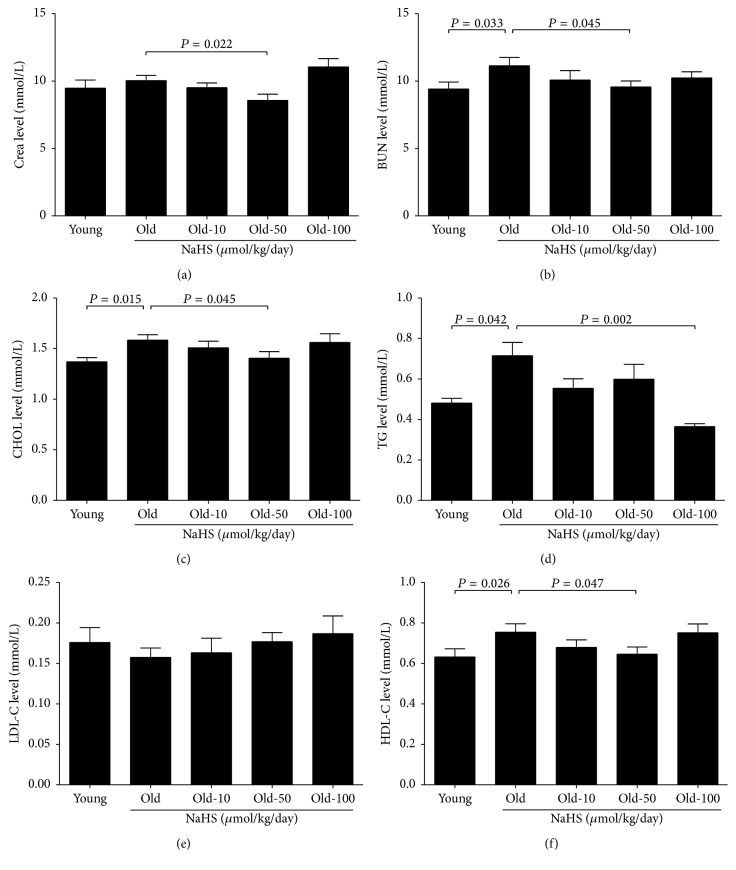
Blood biochemical results for young and old mice. (a) Crea: creatinine. (b) BUN: blood urea nitrogen. (c) CHOL: total cholesterol. (d) TG: triglycerides. (e) LDL-C: low-density lipoprotein. (f) HDL-C: high-density lipoprotein. Values are mean ± SE. *P* < 0.05 was considered significant (young groups, *N* = 12; old groups, *N* = 14).

There was no significant difference in fasting blood-glucose between the young and old groups, and NaHS (10, 50, and 100 *μ*mol/kg/day) treatment changed neither the fasting blood-glucose nor the body weight among old groups (Table 1). Compared with young mice, food and water intake were decreased whereas the urine volume increased in old control mice. Chronic NaHS treatment for 10 weeks did not change the food and water intake (Figures 1(a) and 1(b)) but could decrease the 24-hour urine volume and the contents of urine protein (Figures 1(c) and 1(d)). The maximum effect of NaHS in decreased urine volume was achieved in the 100 *μ*mol/kg/day NaHS treatment group, whereas the 50 *μ*mol/kg/day NaHS treatment group decreased urine protein contents the most (Figures 1(c) and 1(d)). The blood level of Crea and LDL-C did not change (Figures 2(a) and 2(e)), whereas the contents of BUN, CHOL, TG, and HDL-C increased dramatically in old control mice compared with young mice (Figures 2(b), 2(c), 2(d), and 2(f)). Ten weeks of 50 *μ*mol/kg/day NaHS treatment significantly alleviated the increase of Crea, BUN, CHOL, and HDL-C (Figures 2(a), 2(b), 2(c), and 2(f)), and 100 *μ*mol/kg/day NaHS therapy decreased the TG contents (Figure 2(d)). Our results indicate that there is impaired kidney function during ageing, and exogenous H_2_S treatment could partially reverse such impairment.

### 3.2. NaHS Alleviates the Level of Oxidative Stress in the Ageing Kidney

**Figure 3:**
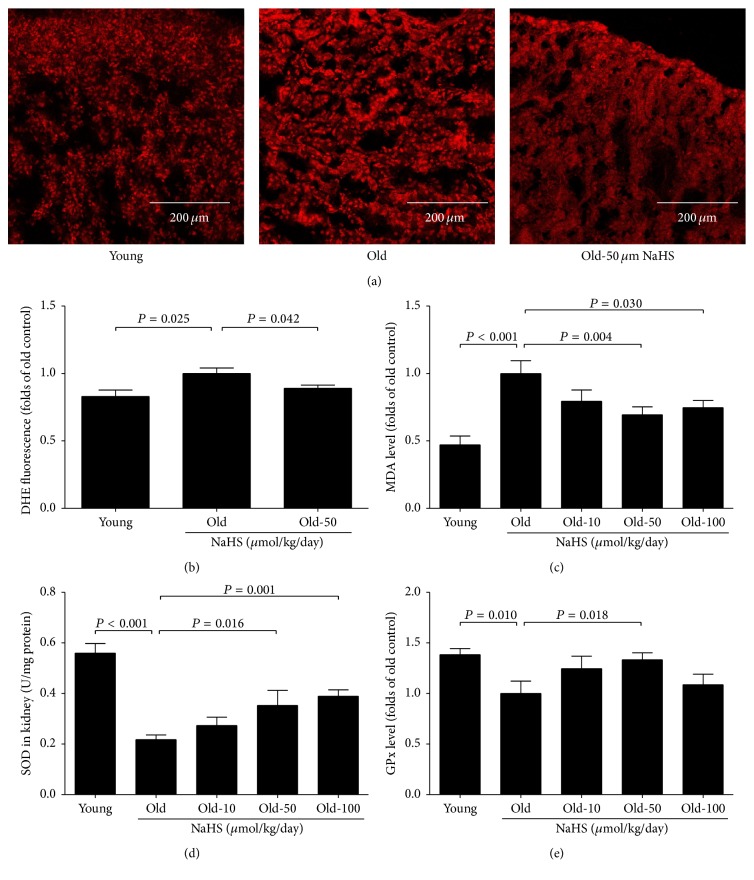
H_2_S donor NaHS protected the ageing kidney from oxidative stress. ((a) and (b)) DHE staining and the DHE fluorescence in the kidney tissue. (c) Malondialdehyde (MDA) levels in the kidney. (d) Total SOD activity renal tissue. (e) Glutathione peroxidase (GPx) levels in the kidney. Values are mean ± SE. *P* < 0.05 was considered significant (young groups, *N* = 6; old groups, *N* = 8).

ROS levels in the kidney were examined after 10 weeks of NaHS therapy. DHE fluorescence intensity and malondialdehyde (MDA) levels were elevated significantly in the old control group compared with young mice, and chronic NaHS treatment could mitigate these changes (Figures 3(a), 3(b), and 3(c)). Accordingly, SOD activity and glutathione peroxidase (GPx) levels were decreased in old mice, and 50 *μ*mol/kg/day NaHS treatment could partially rescue these changes (Figures 3(d) and 3(e)). Our results indicated that the H_2_S donor could protect the ageing kidney from oxidative stress.

### 3.3. Ageing Mice Exhibit Kidney Remodeling and Apoptosis and Chronic NaHS Treatment Mitigates These Processes

**Figure 4:**
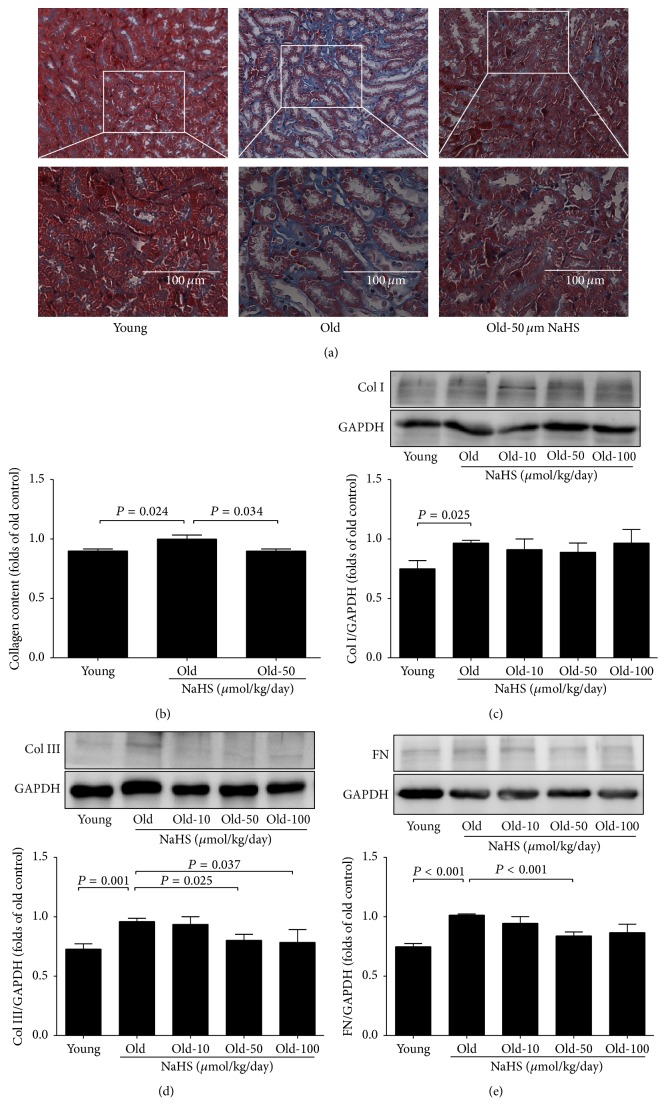
Renal pathological changes of ageing mice. ((a) and (b)) Masson staining of the kidney tissue. (c) Col I and (d) Col III expression in ageing mice (*N* = 8). (e) FN expression in ageing mice (*N* = 10). *P* < 0.05 was considered significant.

Masson staining showed significantly increased interstitial fibrosis compared with young groups, and 10 weeks of 50 *μ*mol/kg/day NaHS treatment could partially reduce collagen deposition (Figures 4(a) and 4(b)). There was higher expression of Col III and FN in the ageing kidney, and this trend became less significant with chronic NaHS treatment (Figures 4(d) and 4(e)). Col I expression was also increased in the ageing kidney, but with no significant difference between the NaHS-treated and nontreated groups (Figure 4(c)). In addition, mRNA levels of Col I, Col III, and FN also increased during ageing (Figure  S1 in Supplementary Material available online at http://dx.doi.org/10.1155/2016/7570489).

**Figure 5:**
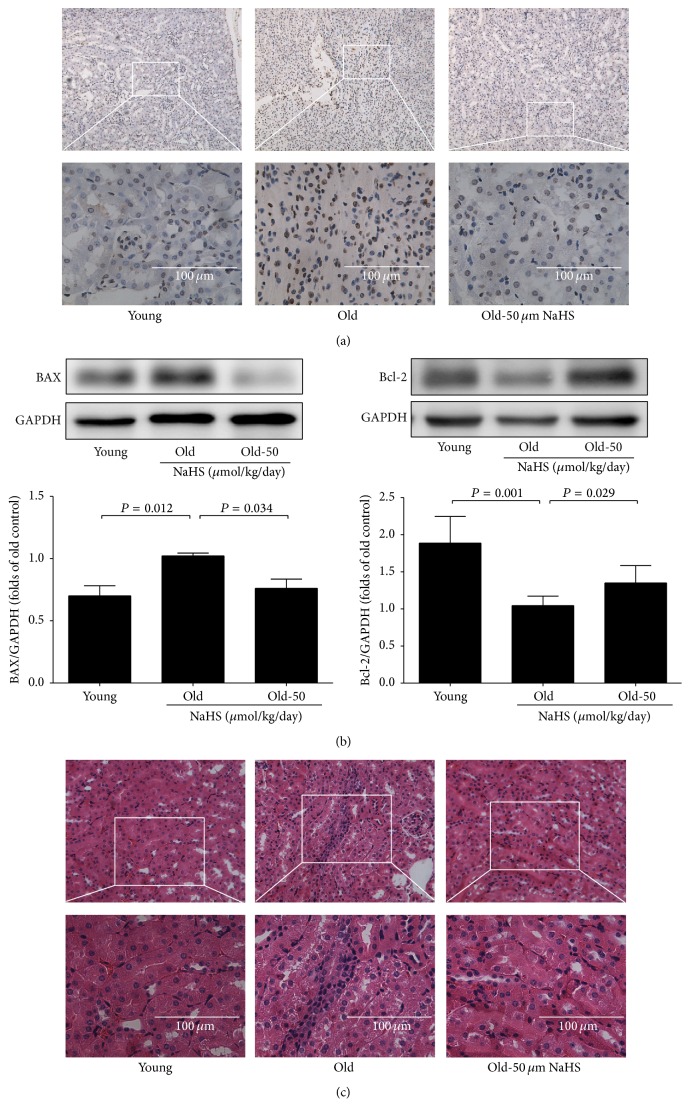
Renal pathological changes of ageing mice. (a) TUNEL staining of the kidney tissue. (b) Bax (*N* = 6) and Bcl-2 (*N* = 7) expression in ageing mice. (c) HE staining of the kidney tissue. Values are means ± SE. *P* < 0.05 was considered significant.

The ageing kidney appeared to have a higher apoptosis level compared with that of the young group, evidenced by increased expression of proapoptotic Bax and decreased expression of antiapoptotic Bcl-2 (Figure 5(b)). TUNEL staining revealed the same changes (Figure 5(a)). HE staining showed tubular dilation and inflammatory infiltration among ageing mice, whereas chronic NaHS therapy could mitigate this damage (Figure 5(c)). Our results indicated that kidney remodeling and apoptosis had occurred in ageing mice, and chronic NaHS treatment could mitigate these processes.

### 3.4. Endogenous Hydrogen Sulfide Production Is Decreased in Ageing Mice; NaHS Treatment Alleviates the Reduction by Increasing the Expression and Activity of Hydrogen Sulfide-Producing Enzymes

**Figure 6:**
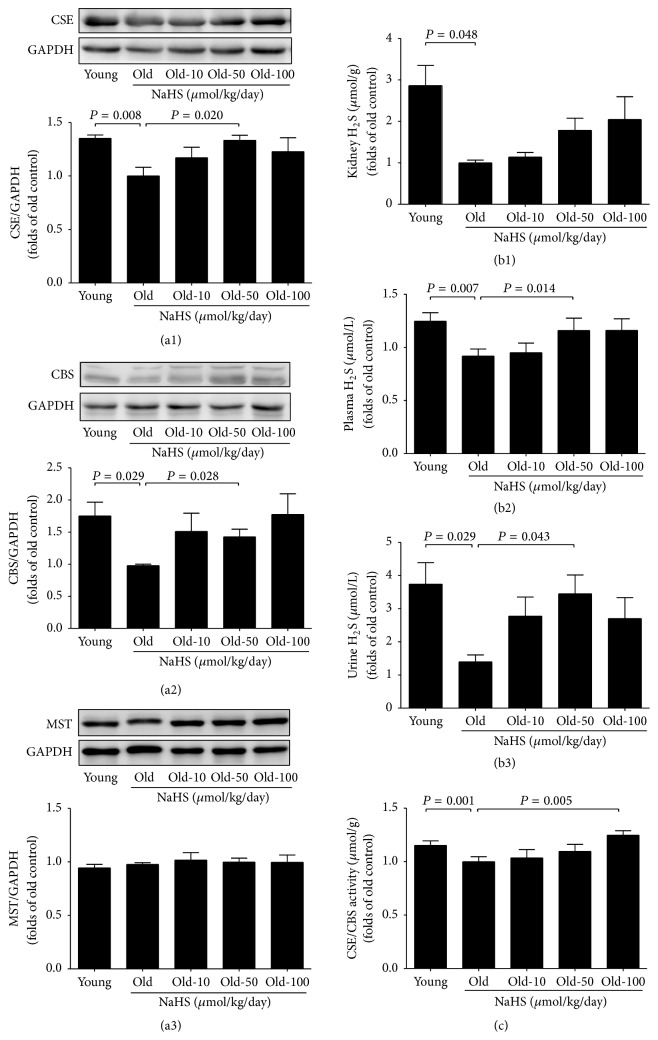
Exogenous H_2_S alleviated ageing-related decreasing of H_2_S production. (a1)–(a3) Kidney expression of CSE (*N* = 11) and CBS decreased significantly, whereas 3-MST kept constant during ageing (*N* = 10). (b1)–(b3) Plasma, urine, and kidney tissue H_2_S levels decreased significantly during ageing and rose again after chronic NaHS treatment. (c) The activity of CSE/CBS in the ageing kidney declined, whereas 100 *μ*mol/kg/day NaHS treatment could mitigate the reduction. Values are means ± SE. *P* < 0.05 was considered significant (*N* = 12).

Compared with young groups, ageing mice showed lower CSE and CBS expression, although 3-MST expression remained unchanged. Ten weeks of NaHS treatments (50 *μ*mol/kg/day) significantly increased the expression of CSE and CBS, but not the 3-MST (Figures 6(a1), 6(a2), and 6(a3)). Plasma, urine, and kidney H_2_S levels decreased significantly during ageing (Figures 6(b1), 6(b2), and 6(b3)), whereas 10 weeks of NaHS (50 *μ*mol/kg/day) treatment alleviated the plasma and urine H_2_S levels (Figures 6(b2) and 6(b3)). H_2_S levels in ageing kidney tissues were also increased to some extent after NaHS treatment, but without statistical significance (Figure 6(b1)). CSE/CBS activity in the ageing kidney decreased, and 100 *μ*mol/kg/day NaHS treatment diminished this reduction (Figure 6(c)).

### 3.5. NaHS Activates SIRT1 in the Ageing Kidney

**Figure 7:**
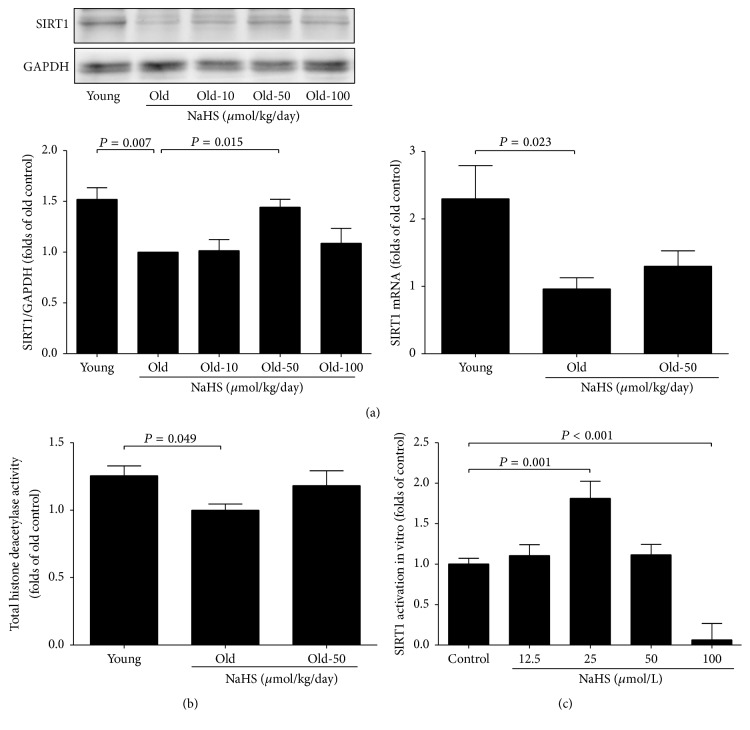
SIRT1 was involved in NaHS-mediated ageing kidney protection. (a) SIRT1 protein and mRNA expressions in the kidney after chronic NaHS treatment for 10 weeks (*N* = 8). (b) Total deacetylase activity exchangeable in the kidney (*N* = 10). (c) NaHS-activated recombinant protein SIRT1 activity in vitro (*N* = 10). Values are mean ± SE. *P* < 0.05 was considered significant.

To determine whether H_2_S protects the ageing kidney through the SIRT1-mediated pathway, the protein and transcriptional levels of SIRT1 were examined. Both the protein expression and mRNA transcription of SIRT1 were decreased in ageing mice compared with young ones, and NaHS (50 *μ*mol/kg/day) treatment could improve SIRT1 protein expression but not the mRNA levels (Figure 7(a)). SIRT1 is a deacetylase, and 10 weeks of NaHS treatment did not affect total deacetylase activity in the ageing kidney (Figure 7(b)), but 25 *μ*mol/L NaHS could directly increase the activity of recombinant SIRT1 protein in vitro (Figure 7(c)). Measurement of SIRT1 activity in the ageing kidney will help to make clear whether chronic NaHS treatment selectively influences SIRT1 activity while total deacetylase activity remains the same.

### 3.6. Effects of Chronic NaHS Treatment on the Expression of Antioxidant-Related Proteins in the Ageing Kidney

**Figure 8:**
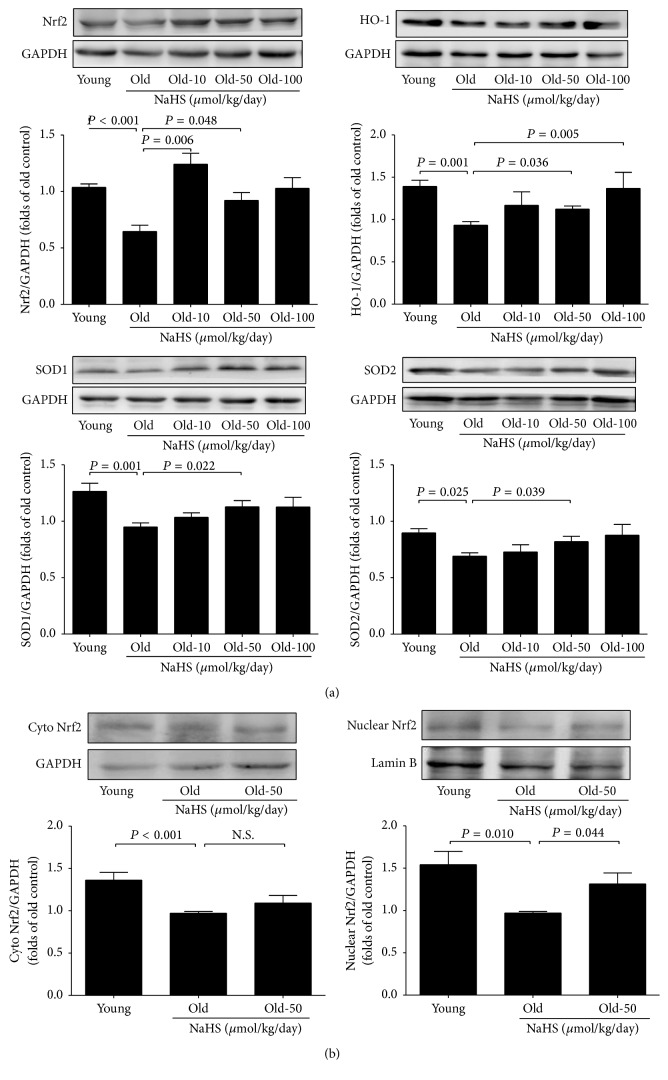
The protective effects of the H_2_S donor on the expression of antioxidant-related proteins in the ageing kidney tissue. (a) The expression change of Nrf2 (*N* = 11), HO-1 (*N* = 10), SOD1 (*N* = 11), and SOD2 (*N* = 10) after 10 weeks of exogenous H_2_S donor. (b) Nrf2 was translocated from cytosol to nucleus, Lamin B was used as nuclear control, and GAPDH was used as cytosol control (*N* = 6). Values are mean ± SE. *P* < 0.05 was considered significant.

The expression of antioxidant proteins in kidney tissue was examined by western blot analysis at the end of chronic NaHS treatment. Ten weeks of NaHS (50 *μ*mol/kg/day) treatment could increase the Nrf2 expression and improve its downstream antioxidative proteins such as HO-1, SOD1, and SOD2 (Figure 8(a)). Moreover, compared with young groups, both the nuclear and cytosol Nrf2 levels were decreased, and NaHS (50 *μ*mol/kg/day) treatment could selectively increase nuclear Nrf2 (Figure 8(b)). As shown in Figure S2A, the Nrf2 nuclear translation in the kidney tissue was insufficient. As shown in Figure S2B, Nrf2 translocation in the NRK-52E cells treated with NaHS (50 *μ*mol/L) was significantly induced from cytosol to nucleus and peaked at 60 min. The translocation of Nrf2 from cytoplasm into nuclear may be one of the protective mechanisms of H_2_S against ageing-related oxidative stress.

## 4. Discussion

In this study, we employed an ageing mouse model to investigate chronic NaHS treatment in the process of kidney senescence. Our work reveals two important findings: (1) lower plasma, urine, and kidney H_2_S levels and reduction of kidney CSE and CBS expression and activity are accompanied with ageing; (2) exogenous administration of H_2_S donor NaHS mitigates ageing-related kidney dysfunction, and the protective effect of NaHS may at least partially relate to improved endogenous H_2_S production and its antioxidative nature.

During ageing, an elevated amount of ROS generated from glycolysis, specifically caused by the defects in the polyol pathway, uncoupling of nitric oxide synthase, xanthine oxidase, and advanced glycation, results in the progressive deterioration of renal function [18]. Emerging data suggest that ROS are related to the pathophysiology of glomerular dysfunction, interstitial fibrosis, and glomerulosclerosis [19, 20]. In streptozotocin-induced diabetic rats, H_2_S therapy (14 *μ*mol/kg/day) improved renal function and decreased glomerular basement thickening, mesangial expansion, and interstitial fibrosis [18]. Our previous study showed that chronic NaHS treatment (30, 60, and 120 *μ*mol/kg/day) significantly reduced ROS levels in the kidney of GK rats [21]. Consistently, we found that chronic NaHS treatment could reduce ROS levels (50 *μ*mol/kg/day) and MDA contents (50 and 100 *μ*mol/kg/day) and increase GPx levels and SOD activity in the kidney of ageing mice. H_2_S takes part in a great variety of physiological and pathophysiological processes because of its antiapoptotic, antioxidative, anti-inflammatory, and proangiogenic activities in mammals, and the reduction of endogenous H_2_S levels has been related to various diseases. Zhou et al. reported that endogenous H_2_S generation and CSE protein expression decreased significantly in the streptozotocin-induced diabetic rat model and that exogenous H_2_S (14 *μ*mol/kg/day) protected against diabetic nephropathy [18]. Our previous study also showed that chronic NaHS (30 *μ*mol/kg/day) treatment might ameliorate diabetic complications of the kidney [21].

In this study, we confirmed intensified interstitial fibrosis in the ageing kidney and observed decreased accumulation of Col III (50 and 100 *μ*mol/kg/day) and FN (at 50 *μ*mol/kg/day) with exogenous NaHS. We also observed increased apoptosis in the ageing kidney, and chronic NaHS treatment could partially reverse this deterioration. Age-related kidney damage might reversely correlate with endogenous H_2_S production. We found that both H_2_S levels in the plasma, urine, and kidney and the kidney expression of CSE and CBS decreased significantly with ageing. As part of our body's antioxidative defense, an insufficient H_2_S system may decrease the overall ability of ROS scavenging and render the kidney to be under oxidative stress. Reasonably, chronic NaHS treatment protects the ageing kidney by ROS scavenging and enhancing endogenous H_2_S production in kidney tissue as reported previously in myocardium [22]. Decreased CBS activity in the ageing kidney may also lead to homocysteine accumulation. Because the latter plays an important role in chronic renal failure [23, 24], further study is needed to explore whether homocysteine metabolism disorder is involved in ageing-related kidney damage and whether exogenous NaHS could help restore the normal metabolic pathway.

Christopher Hine reported that sulfur amino acid restriction could alter H_2_S production and protect the liver from ischemia-reperfusion injury [25], indicating the beneficial role of endogenous H_2_S. SIRT1, which emerges as a major life span regulator, has been widely explored in the cardiovascular system and nervous system, but it is rare in the urinary system. SIRT1 has robust biological effects and can affect metabolic homeostasis and ageing. Consistent with previous studies [26, 27], we found that both the expression and activity of SIRT1 decreased rapidly in the ageing kidney. The reduction of NAD^+^ biosynthesis may be responsible for reduced SIRT1 activity [26]. Here we report an interesting finding that a low concentration of NaHS (25 *μ*mol/L) could directly induce SIRT1 activation in a cell-free system, whereas chronic treatment of NaHS (50 *μ*mol/kg/day) selectively improves the expression but not the activity of SIRT1 in the ageing kidney. SIRT1 also regulates lipid metabolism by manipulating PPAR-*α*, LXR, FXR, and SREBP signals [28, 29]. For example, SIRT1 positively regulates LXR proteins, which act as cholesterol sensor and adjust whole body cholesterol and lipid homeostasis [30]. Our blood biochemical tests showed that chronic NaHS treatment could decrease CHOL and TG. Therefore, H_2_S may modulate liver and renal lipid metabolism through an enhanced SIRT1 signal and contribute to the prevention of renal disease progression.

Nrf2 is an important antioxidative molecule that modulates the expression of antioxidant genes [30] and thus prevents age-related oxidative stress [31, 32]. Here we showed that the kidney Nrf2 expression decreased during ageing and that chronic NaHS treatment could rescue Nrf2 expression (10 and 50 *μ*mol/kg/day) and enhance its nuclear translocation (50 *μ*mol/kg/day). The precise mechanism of excessive oxidative stress in ageing is not clear, but the decreased expression of Nrf2/antioxidant stress element-linked antioxidant genes is thought to play a role [32–34]. We found that chronic NaHS treatment could activate Nrf2 downstream genes such as HO-1, SOD1, and SOD2, which consequently enhanced resistance to oxidative stress in the ageing kidney. Taken together, our data support the idea that H_2_S plays a crucial role in protecting the ageing kidney from antifibrosis and antiapoptosis through the regulation of redox homeostasis.

## 5. Conclusion

In summary, we demonstrated that 3-MST was expressed in the ageing kidney and that endogenous H_2_S levels decreased because of the impaired expression of H_2_S-producing enzymes. Chronic exogenous H_2_S treatment could protect the ageing kidney by reducing oxidative stress, decreasing collagen deposition, and enhancing Nrf2 nuclear translocation, as well as increasing endogenous H_2_S production.

## Supplementary Material


*Immunofluorescence and cell treatment.* D-galactose (D-gal) was purchased from Sigma (German). Sub-acute senescence was induced in renal tubular epithelial cells (NRK-52E) through incubating cells with 50 mmol/L D-gal [36] for 48 h and treated with 50 μmol/L NaHS for different duration. Cells were then fixed at 0 min, 15 min, 30 min, 60 min, 120 min after NaHS treatment in immunol staining fix solution (Beyotime Biotechnology, Nanjing), permeabilized with 10% triton and blocked with 5% bovine serum albumin for 60 min at room temperature. They were then incubated overnight with Nrf2 antibody (1 : 100, Proteintech, China) at 4°C, washed with PBS (5 min, three times) and incubated with anti-rabbit Alexa Fluor 488 (1 : 100, Thermo Fisher Scientific, Waltham, MA, USA) for 1 hour at room temperature and washed again. Nucleus was stained with DAPI (Beyotime Biotechnology, Nanjing) for 5 min. Optimal cutting temperature compound-embedded kidney sections (7 μm) was permeabilized and antigen renovated. Then they were treated by the same method of cell samples, mounted with anti-fade mounting medium (Beyotime Biotechnology, Nanjing), and observed by laser confocal microscope (Zeiss LSM710).

## Figures and Tables

**Table 1:** Results are means ± SE. Old groups were treated with a variety of NaHS (0, 10, 50, and 100 *μ*mol/kg/day) treatments for 10 weeks. ^*∗*^ *P* < 0.05 and ^*∗∗*^ *P* < 0.01 compared with old control (young group, *N* = 14; old groups, *N* = 18).

	Old	Young	Old-10	Old-50	Old-100
Body weights (bw) (g)	29.26 ± 3.03	20.88 ± 02.30^*∗∗*^	2939 ± 0.46	27.37 ± 1.7	27.17 ± 2.69
Fasting plasma glucose (mmol/L)	3.8429 ± 0.4315	3.9143 ± 0.4140	3.9600 ± 0.5771	3.8833 ± 0.5419	4.1200 ± 0.5762
Heart mass/bw	0.0052 ± 0.0003	0.0058 ± 0.0008	0.0062 ± 0.0008^*∗∗*^	0.0061 ± 0.0008^*∗∗*^	0.0058 ± 0.0007^*∗∗*^
Liver mass/bw	0.0446 ± 0.0041	0.0464 ± 0.0046	0.0465 ± 0.0037	0.0457 ± 0.0017	0.0454 ± 0.0024
Left kidney mass/bw	0.0064 ± 0.0007	0.0066 ± 0.0011	0.0067 ± 0.0007	0.0072 ± 0.0003^*∗∗*^	0.0073 ± 0.0004^*∗∗*^
Right kidney mass/bw	0.0065 ± 0.0007	0.0059 ± 0.0016	0.0069 ± 0.0008	0.0074 ± 0.0004^*∗∗*^	0.0073 ± 0.0004^*∗*^
