# Supplementary material for: Protective Effects of Hydrogen Sulfide in the Ageing Kidney
Source: Oxid Med Cell Longev. 2016 Nov 1;2016:7570489. doi: 10.1155/2016/7570489 (PMC5108860; doi:10.1155/2016/7570489)
Supplement: Supplementary file 1 — Immunofluorescence and cell treatment. D-galactose (D-gal) was purchased from Sigma (German). Sub-acute senescence was induced in renal tubular epithelial cells (NRK-52E) through incubating cells with 50 mmol/L D-gal [36] for 48 h and treated with 50 μmol/L NaHS for different duration. Cells were then fixed at 0 min, 15 min, 30 min, 60 min, 120 min after NaHS treatment in immunol staining fix solution (Beyotime Biotechnology, Nanjing), permeabilized with 10% triton and blocked with 5% bovine serum albumin for 60 min at room temperature. They were then incubated overnight with Nrf2 antibody (1 : 100, Proteintech, China) at 4°C, washed with PBS (5 min, three times) and incubated with anti-rabbit Alexa Fluor 488 (1 : 100, Thermo Fisher Scientific, Waltham, MA, USA) for 1 hour at room temperature and washed again. Nucleus was stained with DAPI (Beyotime Biotechnology, Nanjing) for 5 min. Optimal cutting temperature compound-embedded kidney sections (7 μm) was permeabilized and antigen renovated. Then they were treated by the same method of cell samples, mounted with anti-fade mounting medium (Beyotime Biotechnology, Nanjing), and observed by laser confocal microscope (Zeiss LSM710). [file 7570489.f1.pdf]

Supplementary Material

*Immunofluorescence and cell treatment.*

D-galactose (D-gal) was purchased from Sigma (German). Sub-acute senescence was induced in renal tubular epithelial cells (NRK-52E) through incubating cells with 50 mmol/L D-gal [36] for 48h and treated with 50 μmol/L NaHS for different duration. Cells were then fixed at 0 min, 15 min, 30 min, 60 min, 120 min after NaHS treatment in immunol staining fix solution (Beyotime Biotechnology, Nanjing), permeabilized with 10% triton and blocked with 5% bovine serum albumin for 60 min at room temperature. They were then incubated overnight with Nrf2 antibody (1 : 100, Proteintech, China) at 4°C, washed with PBS (5 min, three times) and incubated with anti-rabbit Alexa Fluor 488 (1 : 100, Thermo Fisher Scientific, Waltham, MA, USA) for 1 hour at room temperature and washed again. Nucleus was stained with DAPI (Beyotime Biotechnology, Nanjing) for 5 min.

Optimal cutting temperature compound--embedded kidney sections (7 μm) was permeabilized and antigen renovated. Then they were treated by the same method of cell samples, mounted with anti-fade mounting medium (Beyotime Biotechnology, Nanjing), and observed by laser confocal microscope (Zeiss LSM710).

Figure S1

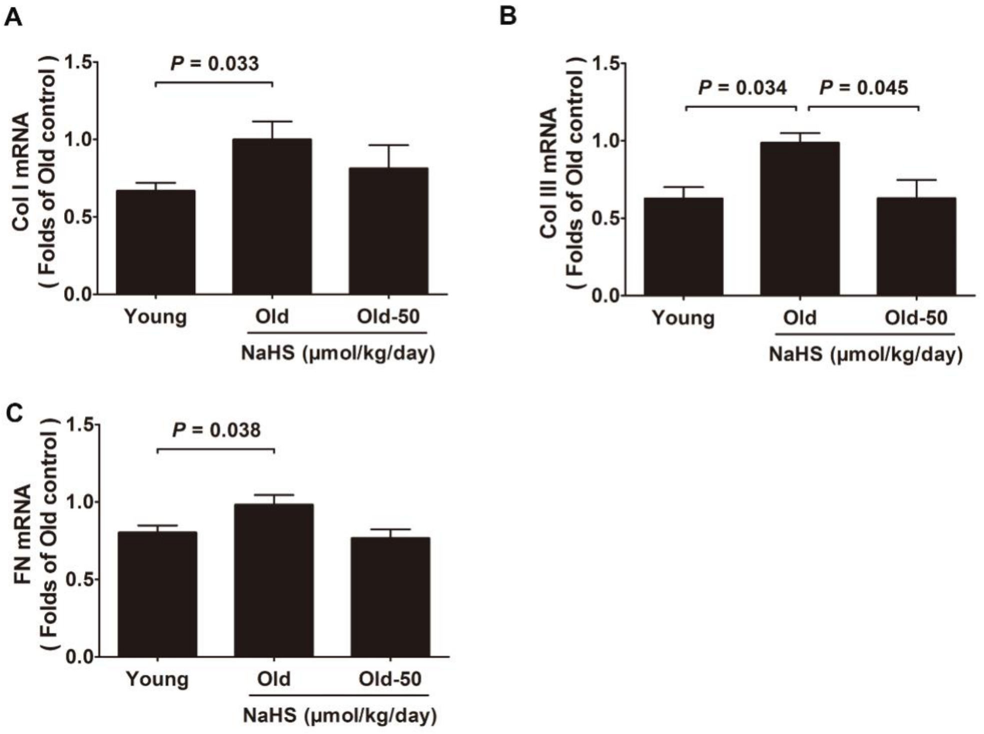


**Figure S1:** Regulation of $H_2S$ on renal fibrosis related genes. A -C. The mRNA levels of Col I, Col III, and FN were increased significantly, while chronic NaHS treatment could partly reverse the increase (N=11). Values are mean ± SE. $P < 0.05$ was considered significant.

Figure S2

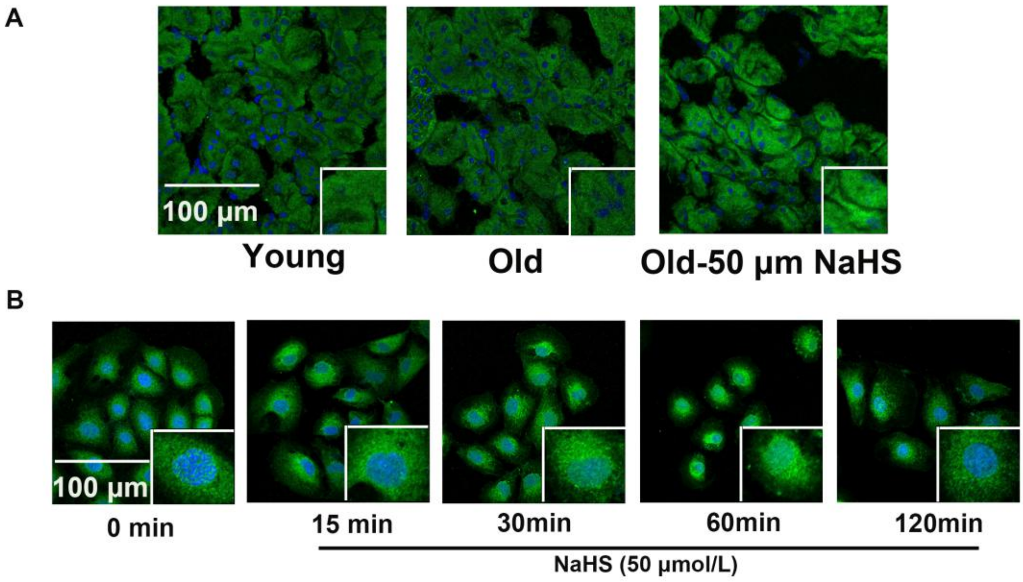


**Figure S2** The protective effects of the $H_2S$ donor on the expression of Nrf2 in ageing kidney tissue and NRK-52E cells. Green is Nrf2 immunofluorescence staining and blue is nuclear staining. The bottom right corner picture showed details with higher magnification. A. Nrf2 was translocated from cytosol to nucleus in kidney tissue with NaHS treatment (N=3). B. Nrf2 was translocated from cytosol to nucleus in NRK-52E cells, Lamin B was used as the nuclear control, and GAPDH was used as the cytosol control (N=3). Values are mean ± SE. $P < 0.05$ was considered significant.
